# Solving Vibronic Dynamics in Electron Continuum

**DOI:** 10.1021/acs.jctc.3c01217

**Published:** 2024-02-07

**Authors:** Martina Ćosićová, Jan Dvořák, Martin Čížek

**Affiliations:** Faculty of Mathematics and Physics, Institute of Theoretical Physics, Charles University, V Holešovičkách 2, 180 00 Prague, Czech Republic

## Abstract

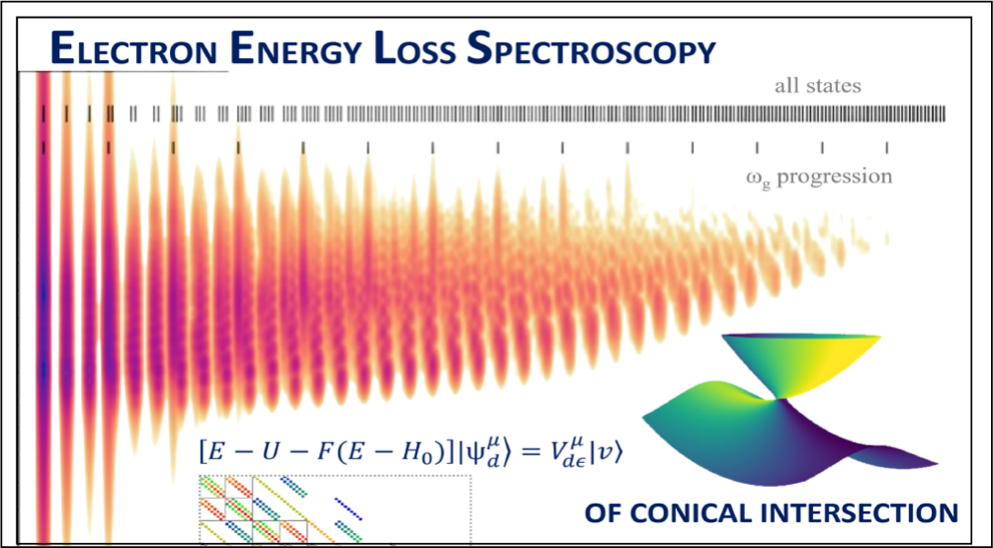

We present a general two-dimensional model of conical intersection between metastable states that are vibronically coupled not only directly but also indirectly through a virtual electron in the autodetachment continuum. This model is used as a test ground for the design and comparison of iterative solvers for resonance dynamics in low-energy electron–molecule collisions. Two Krylov-subspace methods with various preconditioning schemes are compared. To demonstrate the applicability of the proposed methods on even larger models, we also test the performance of one of the methods on a recent model of vibrational excitation of CO_2_ by electron impact based on three vibronically coupled discrete states in continuum (Renner–Teller doublet of shape resonances coupled to a sigma virtual state) including four vibrational degrees of freedom. Two-dimensional electron energy-loss spectra resulting from electron–molecule scattering within the models are briefly discussed.

## Introduction

Despite a long history of investigations (see for example reviews^1−5^), the collisions of low-energy electrons with molecules still represent a fascinating and challenging field of study. By low energy we mean here the energy below the electronic-excitation threshold, i.e., the energy that does not exceed few units of electronvolts. Even these low energies lead to many interesting phenomena like the appearance of sharp structures in cross sections^5,6^ or the possibility to select dissociation into different anionic fragments by tuning the energy.^7,8^ This topic is both interesting for practical applications^9^ and challenging for the theory even for small polyatomic molecules.^10−16^

In this paper, we will focus on the process of vibrational excitation in collision of an electron *e*^–^ with a molecule *M* initially in a vibrational state |*v*_*i*_⟩
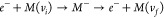
1mediated by one or several metastable anion states *M*^–^. After the process, the molecule is left in the final vibrational state |*v_f_*⟩. The total energy *E* during the collision is conserved
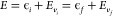
2where ϵ are electron energies and *E_v_* the energies of vibrational states of the molecule for the initial and final states before and after the collision. This process is closely related to the process of photodetachment of an electron *e*^–^ from a molecular anion *M*^–^
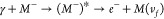
3with initial energy *E* of the system defined now by the energy of the photon γ shone on the anion to excite it to the state (*M*^–^)*. The dynamics of both of these processes is driven by potential energy of states of the negative molecular ion and their widths for decay into electronic continuum channels.^17^ The energies of the released electrons are sensitive to the relative position of the anion and the neutral molecular states, and the selection rules are different than for the radiative transitions.^18−20^

The goal of this paper is to advance the detailed theory of the dynamics of electron detachment from anions in such processes. In the development of the theoretical methods, we keep in mind the description of experiments that study in detail the energies of released electrons,^17,21,22^ and in particular, we calculate the two-dimensional electron energy-loss spectrum (2D EELS) for our model. The 2D electron loss spectroscopy was pioneered by Currell and Comer^23,24^ and further developed by Allan and collaborators.^25^ To date, a dozen of high-resolution spectra for different molecules have been measured^17,25−32^ but the detailed understanding of such spectra for polyatomic molecules is mostly lacking.

In this paper, we present and test a general scheme for solving the nuclear dynamics of the negative ion formed in the collision of an electron with a polyatomic molecule. The scheme is tailored for a class of models that are inspired by the pseudo-Jahn–Teller model of Estrada, Cederbaum, and Domcke^33^ with modifications meant to make it a more realistic model of real molecules. This approach combines a model of vibronic coupling of several anionic states expanded in low-order polynomials in vibrational coordinates close to equilibrium geometry of the neutral molecule with a projection-operator approach to include the interaction of the anion discrete states with the electronic continuum. The model is rather flexible in adding states and vibrational degrees of freedom and the present scheme has been used to produce the results in our previous works on CO_2_.^34−36^ In these papers, we did not explain the methods and their performance in detail, a gap that is meant to be filled by this work.

We start the “Theory” section by reviewing the projection-operator approach to the dynamics of vibrational excitation in electron collisions with molecules. We then proceed by reminding the model of Estrada et al.^33^ and propose its generalization by including the vibronic coupling through the electron continuum in addition to the direct vibronic coupling present in the original model. This section is concluded by explaining the representation of the wave function components and the Hamiltonian in a basis constructed from neutral vibrational states. In “Krylov-Subspace Iteration Methods“ section, we first briefly introduce (the details are in the Appendix) used iteration methods and preconditioning schemes, and then we discuss their performance for the models. The section “Discussion of Resulting Spectra for Test Models” is devoted to a brief description of the obtained 2D spectra for the models, and we conclude by summarizing the results in the “Conclusions” section.

## Theory

The vibrational and resonance dynamics in electron–molecule collisions has been studied theoretically for a long time (see, for example, one of the review papers^2,4,37,38^). The direct brute-force approach is only tractable for small molecules^39^ or for small deformations.^40^ A number of approximate schemes have therefore been developed: Born approximation, adiabatic-nuclei approximation, zero/effective range, or semiclassical approaches. In the present work, we focus on the development of the numerical schemes for the projection-operator approach based on the existence of an intermediate anion state (or states) that is responsible for the coupling of the electronic and vibrational motion. The approach is often used in its approximate form—the local complex potential approximation, but it is known to fail in predicting interesting phenomena like Wigner cusps or vibrationally excited Feshbach resonances. The nonlocal approach is well-developed for diatomic molecules^4,41^ but the attempts to use it for polyatomic molecules are scarce (see for example the paper by Ambalampitiya and Fabrikant^42^). In addition to bringing more degrees of freedom, the polyatomic molecules also exhibit interesting features like vibronic coupling of resonances, conical intersections, and exceptional points.^43,44^ Here, we follow the work of Estrada, Cederbaum, and Domcke^33^ (ECD86) and extend it to more general form of model functions and vibronic coupling. We start by presenting basic formulas resulting from the projection-operator formalism (for comprehensive review of the approach, see the paper by Domcke^4^). Then, we narrow the model to two vibrational degrees of freedom and two vibronically coupled discrete states.

### Nonlocal Model for Multiple Discrete States in Continuum

The main idea of the nonlocal discrete state in continuum model is the assumption that the coupling of the electronic and vibrational degrees of freedom in the electron–molecule collision is mediated by one or a few discrete states and after their removal from the electronic continuum using projection-operator formalism of Feshbach,^45^ the electronic basis consisting of the discrete states and the orthogonalized continuum is diabatic. The vibrational excitation or dissociative attachment then proceeds through capture in the discrete state.

We define the projection operator
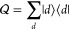
4as a sum over a set of discrete states |*d*⟩ and the complementary operator
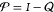
5projecting on the background continuum. The basis in the background part can be chosen as the states that solve the background scattering problem
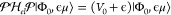
6Here, *V*_0_(*q⃗*) is the potential energy surface of the neutral molecule, i.e., the energy of the ground electronic state |Φ_0_⟩ as a function of the positions of the nuclei *q⃗*. Since we consider only low-energy electron scattering below the threshold for the electronic excitation of the molecule, the state Φ_0_ is fixed, and we will further omit it from the notation. The electron continuum states |ϵμ⟩ are thus uniquely described by the electron energy ϵ and some other quantum numbers collectively denoted by μ (typically angular momentum). All states |*d*⟩ and |ϵμ⟩ thus form an orthogonal basis
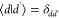
7
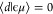
8
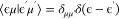
9

The electronic Hamiltonian in the -space is described by a matrix
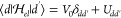
10where all matrix elements depend on the molecular geometry, i.e., positions of nuclei *q⃗*. The diagonal elements *V*_*d*_(*q⃗*) = *V*_0_(*q⃗*) + *U*_*dd*_(*q⃗*) represent the diabatic discrete-state potentials and the off-diagonal part *U*_*dd*′_(*q⃗*) the direct vibronic coupling among the states. The coupling between the discrete state |*d*⟩ and the continuum |ϵμ⟩ is described by the coupling elements
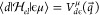
11These elements represent the vibronic coupling^1^ between the discrete state and the continuum, and they also lead to the second-order vibronic coupling among the discrete states mediated by the continuum as described below.

This way, we parametrized the matrix elements given by eqs 6, 10, and 11 of the Hamiltonian  for the electron scattering from the molecule for each fixed position of the nuclei *q⃗* by functions *V*_0_(*q⃗*), *U*_*dd*′_(*q⃗*), and *V*_*d*ϵ_^μ^(*q⃗*). To describe the electron scattering from the molecule including the vibronic dynamics, we start from the definition of the vibrational states |*v*⟩ of the target neutral molecule
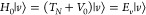
12where *T*_*N*_ is the kinetic-energy operator for the nuclei and *v* is a set of quantum numbers that uniquely determine the vibrational states with energy *E*_*v*_. It can be shown (see for example the review of Domcke^4^) that the vibronic motion of the anion is described by the effective Hamiltonian
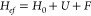
13which is the matrix in the indices *d*, *d*′ and the operator in the space of vibrational degrees of freedom. In the equation above, *H*_0_ is the Hamiltonian operator for the vibrations of the molecule multiplied by unity matrix δ_*dd*′_ in the discrete-state indices, *U* is the matrix with the elements *U*_*dd*′_ defined above, and the operator *F* describes the dynamical coupling of the discrete-state space to the electronic continuum
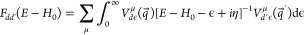
14where η is a positive infinitesimal. This operator is also a matrix in the discrete-state indices and a nonlocal operator in the nuclear coordinate *q⃗*.

The discrete-state contribution to the *T*-matrix for vibrational excitation by electron scattering in a continuum state |ϵ_*i*_ μ_*i*_⟩ from the initial vibrational state *v*_*i*_ to final state *v*_*f*_ and leaving in continuum state |ϵ_*f*_ μ_*f*_⟩ is given by^33^
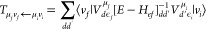
15and is closely related to the integral cross section for the vibrational excitation event
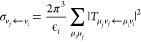
16Finally, to simulate the full 2D electron energy-loss spectra, we have to collect vibrational excitation cross sections for all accessible final states
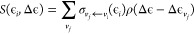
17where ρ(ϵ) is the resolution function of the spectrometer (simulated here with a Gaussian function with full width at half maximum equal to 10 meV, which is comparable to the values in the ref (25)). The energy loss Δ*ϵ*_*v*_*f*__ = *E*_*v*_*f*__ – *E*_*v*_*i*__ = ϵ_*i*_ – ϵ_*f*_ in each term in eq 17 is fixed by the energy conservation. The function *S*(ϵ_*i*_, Δϵ) gives the full experimental information in the electron energy-loss spectroscopy except for the angular resolution that can also be included,^36^ but it is not of interest in the present paper.

### Pseudo-Jahn–Teller Model of Estrada et al.

The model by Estrada et al.^33^ assumes a molecule with an Abelian group of symmetry. They consider two discrete states *d* = 1, 2 that transform according to different irreducible representations of the symmetry group and are coupled vibronically through a nontotally symmetric vibrational mode *q*_*u*_. They also consider excitation of another totally symmetric mode *q*_*g*_, so that the geometry of the molecule within the model is described by a vector *q⃗* = (*q*_*g*_,*q*_*u*_). The symmetry then dictates the structure of matrix *U*
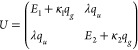
18This is a completely general form of the dependence of matrix *U* on coordinates when the terms are restricted up to the first order in *q⃗* and the symmetry requirements are taken into account. Similarly, we can expand the matrix of the discrete-state-continuum coupling. For simplicity, we consider only two partial waves |ϵμ⟩ for μ = *e*, *o*, representing one even and one odd linear combination of partial waves coupled to the discrete-state space. In principle, we could consider more partial waves but they could be decoupled from the problem by a unitary transformation, thus grouping partial waves into effective channels with the number of channels not exceeding the dimension of the -space.^46^ Estrada et al.^33^ considered the coupling matrix *V*_*d*ϵ_^μ^ independent of the nuclear coordinates (we are going to lift this restriction in the next section). The symmetry selection rules then forbid the coupling *V*_*d*ϵ_^μ^ between the different symmetry of discrete state *d* and partial wave μ. We therefore assume that the discrete state *d* = 1 has even symmetry as the μ = *e* partial wave and *d* = 2 has the symmetry of the odd partial wave μ = *o*. Using eq 14, we then see that only the diagonal matrix elements *F*_11_(ϵ) and *F*_22_(ϵ) of the level-shift operator are nonzero. They can be generated from their imaginary parts (widths)
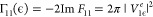
19
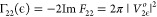
20by means of the integral transform  defined as (compare eq 14)
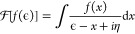
21This transform can be worked out analytically for the assumed form of the widths
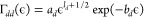
22(see Berman et al.^47^). To complete the model description, we must give the vibrational Hamiltonian *H*_0_ of the neutral molecule. The model simply assumes harmonic vibrations
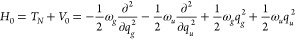
23

The vibrational eigenstates |*v*⟩ satisfying eq 12 with this harmonic Hamiltonian can be numbered by two quantum numbers *ν* = (*n*_*g*_, *n*_*u*_) and the vibrational energies are given by standard harmonic oscillator formula
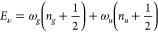


**Figure 1:**
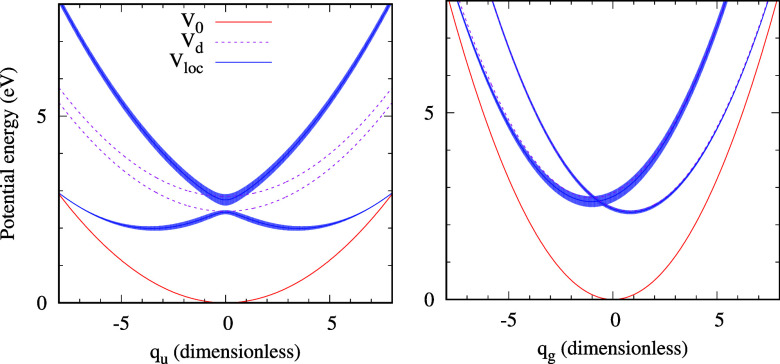
Sections through the potential energy surfaces in the *q*_*g*_ = 0 (left) and *q*_*u*_ = 0 (right) planes for the ECD86 model. Blue-shaded areas give the position and width of the fixed nuclei electronic resonance. See the text for more details.

**Figure 2:**
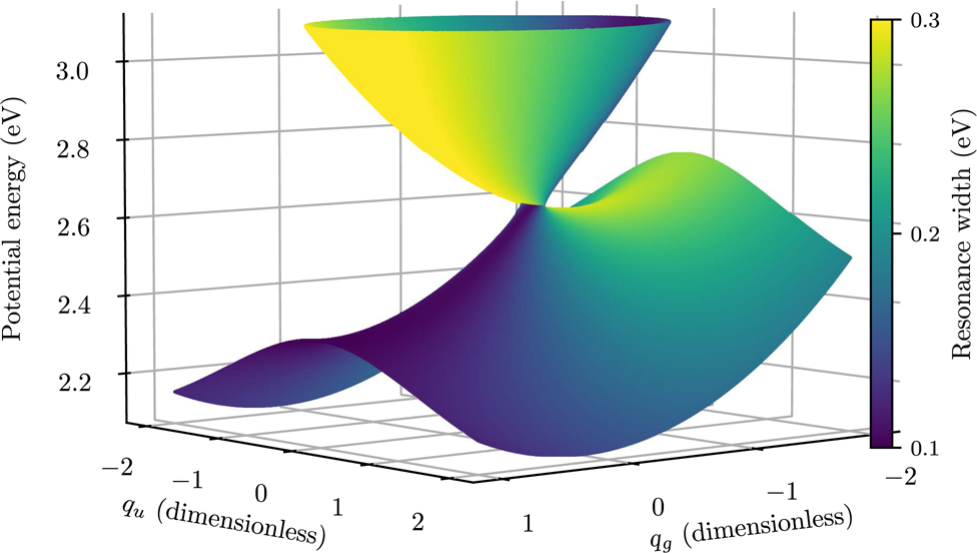
Perspective view of the potential energy manifold for the ECD86 model. The width (inverse lifetime) is marked by the color scale.

The numerical values of ω_*i*_ and the parameters defining direct coupling matrix *U* and discrete-state-continuum matrix *V*_*e*_ for the model studied in Estrada et al.^33^ and used here for testing are given in Table 1 (for dimensionless lengths and energies in units of eV). Note that several variants of the model were used by Estrada et al.^33^ Here, we study only the most complex form of the model with the values of parameters, as given in the table. To visualize the character of the model, we show the one-dimensional sections through the model potentials in Figure 1. The functions shown are *V*_0_(*q⃗*), *V*_*d*_(*q⃗*) = *V*_0_(*q⃗*) + *U*_*dd*_(*q⃗*), and the local complex potential , obtained from the pole of the fixed-nuclei K matrix, which has to be located iteratively.^35^ The perspective view of the local complex potential Re *V*_loc_ colored by values of −2 Im *V*_loc_ is also shown in Figure 2.

**Table 1:** Values of the Parameters Describing the Pseudo-Jahn–Teller Model of Estrada et al.^33^^,^^a^

parameter	value	parameter	value
ω_*g*_	0.258	ω_*u*_	0.091
*E*_1_	2.45	*E*_2_	2.85
κ_1_	–0.212	κ_2_	0.254
λ	0.318		
*a*_1_	0.086	*a*_2_	0.186
*b*_1_	0.833	*b*_2_	0.375
*l*_1_	2	*l*_2_	1

a The dimension of *a*_*d*_ is eV^1/2–l_*d*_^ (*l*_*d*_ is dimensionless), *b*_*d*_ are in eV^–1^, and the remaining parameters are in eV.

### Generalized Model with Vibronic Coupling with Continuum States

The vibronic model above assumes the most simple structure of the discrete-state-continuum coupling matrix *V*_ϵ_ = {*V*_*d*ϵ_^μ^} with row index *d* and column index μ
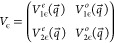
24where , , and *V*_1ϵ_^*o*^(*q⃗*) = *V*_2ϵ_^*e*^(*q⃗*) = 0. We go one step beyond the approximation of the coupling matrix by constant terms, and we expand the matrix to the first order in the normal vibrational coordinates. This generalization is very useful in the description of the interaction of resonances through the electronic continuum that is switched off in the equilibrium geometry but becomes nonzero with deformation as, for example, in the pyrrole molecule.^48^ This feature was also an important ingredient of the model for the CO_2_ molecule.^34,35^ We will further assume that the dependence of *V*_*d*ϵ_(*q⃗*) = *f*(ϵ)*g*(*q⃗*) on the electron energy ϵ and the normal coordinates is separable. Taking into account the symmetry of the system, we get
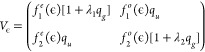
25where the terms that couple a discrete state to the partial wave of different symmetry must be odd functions of *q*_*u*_. We see that half of the total number of 12 terms (up to first order in *q⃗*) in the coupling matrix are zero due to the symmetry. For the purposes of the testing of the numerical methods, we choose the same form of the energy dependence as in the original model
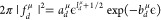
26with the values of the parameters given in Table 2.

**Table 2:** Values of the Parameters Describing the Generalization of the Model^a^

parameter	value	parameter	value
*a*_1_^*e*^	0.07	*a*_2_^*e*^	0.1
*b*_1_^*e*^	0.25	*b*_2_^*e*^	0.5
*l*_1_^*e*^	0	*l*_2_^*e*^	0
*a*_1_^*o*^	0.186	*a*_2_^*o*^	0.15
*b*_1_^*o*^	0.375	*b*_2_^*o*^	0.8
*l*_1_^*o*^	1	*l*_2_^*o*^	1
λ_1_	0.2	λ_2_	0.1

a The dimension of *a*_*d*_^μ^ is eV^1/2–*l*_*d*_^μ^^ (*l*_*d*_^μ^ is dimensionless), *b*_*d*_^μ^ are in eV^–1^, and the remaining parameters are in eV.

Using eq 14, we see that the structure of the nonlocal level-shift operator *F*(*E* – *H*_0_) is much richer
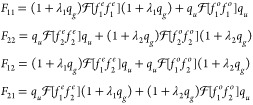
27where we used the integral transform (21) again. The ordering of the terms that depend on *q*_*i*_ with respect to  must be kept because we substitute the operator ϵ = *E* – *H*_0_, which does not commute with the normal coordinates. The off-diagonal terms like *F*_12_ and *F*_21_ that introduce coupling of the discrete states through the continuum have been studied before in the local complex potential approximation (see for example refs (49−51)) among resonances of the same symmetry. A coupling of resonances of different symmetry requires dependence on vibrational coordinates. To the best of our knowledge, our work is the first one to include the full nonlocal form.

**Figure 3:**
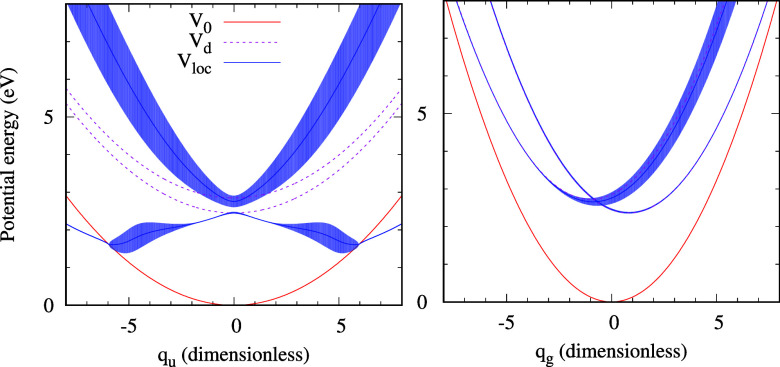
Sections through the model potentials in the *q*_*g*_ = 0 (left) and *q*_*u*_ = 0 (right) planes for the new model. See Figure 1 for more details.

**Figure 4:**
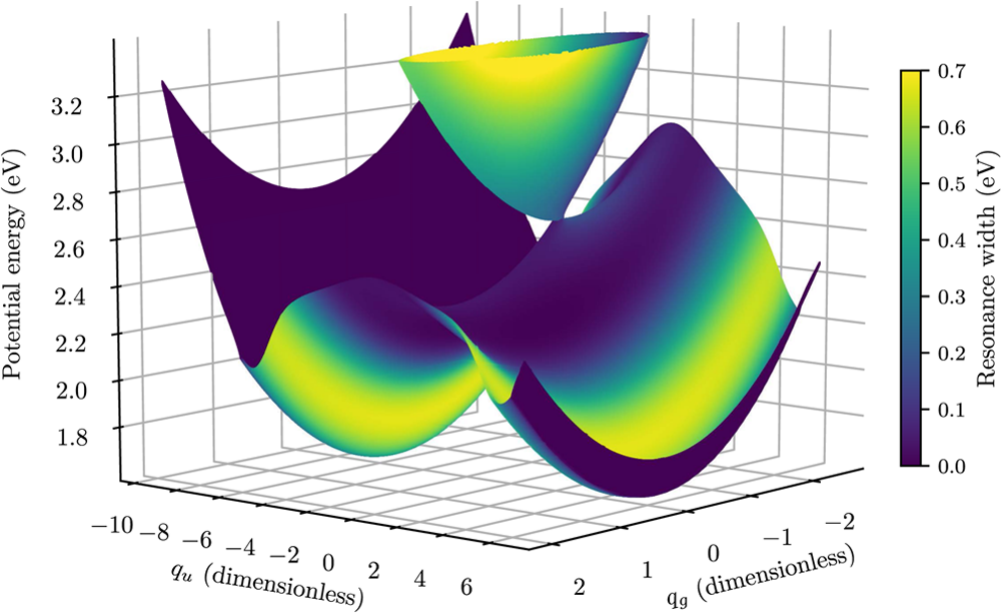
Perspective view of the potential energy manifold for the new model, colored by the width of the resonance.

The potentials for the new generalized model are visualized in Figures 3 and 4. Note that the structure of such conical intersections in continuum has been investigated by Feuerbacher et al.^43,44^ In accordance with their findings, the potential manifolds shown in Figures 2 and 4 do not intersect in a single point like regular conical intersections but in a line segment bounded by two exceptional points, where not only the real but also the imaginary part of the two potentials is identical. The form of our model as given by eq 27 is more general than the expansion investigated by Feuerbacher et al.^43,44^ because they studied a linear coordinate expansion of the width function Γ whereas we prescribe the linear expansion of the coupling matrix *V*_ϵ_, which is more natural for the subsequent treatment of the dynamics. In our case, the linear form of the coupling matrix also produces quadratic terms in widths in eq 27. When the quadratic terms are omitted, we recover the form used in Feuerbacher et al.^43,44^ However, we cannot omit these terms in the dynamics since it would distort the unitarity of the S matrix.

### Numerical Representation of the Dynamics

For the numerical solution of the dynamics, we expand wave function components in the harmonic oscillator basis |ν⟩ = |*n*_*g*_, *n*_*u*_⟩ associated with the model Hamiltonian of the neutral molecule (23). We first rewrite eqs 15 and 16 for the cross section as
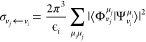
28where we defined auxiliary wave functions |Φ_*v*_^μ^⟩ with components
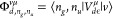
29and with energy according to the conservation law (2), i.e., ϵ = *E* – *E*_*v*_. The anion wave function |Ψ_*v*_*i*__^μ_*i*_^⟩ satisfies
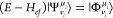
30In the harmonic oscillator basis, this equation represents a system of linear equations for unknown components of the discrete-state wave function
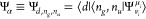
31where we introduced a compound index α ≡ (*d*, *n*_*g*_, *n*_*u*_). Using this notation, the matrix of this system *A*_α,α′_ reads
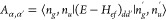
32and the scalar product in eq 28 can be written as the sum over the components of Φ_α_^*v*_*f*_μ_*f*_^ and Ψ_α_
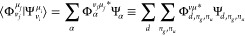
33We cut off the basis in each dimension, keeping the states |*n*_*g*_⟩ for *n*_*g*_ = 0,1, ···, *N*_*g*_–1 and |*n*_*u*_⟩ for *n*_*u*_ = 0,1, ···, *N*_*u*_–1. The states Ψ_α_ are thus represented by *N* = 2*N*_*g*_*N*_*u*_ component vectors and *A* is a *N* × *N* matrix. Typical values used in the following calculations, *N*_*u*_ = 100, *N*_*g*_ = 50, result in converged cross sections, as we checked by varying these parameters.

**Figure A1:**
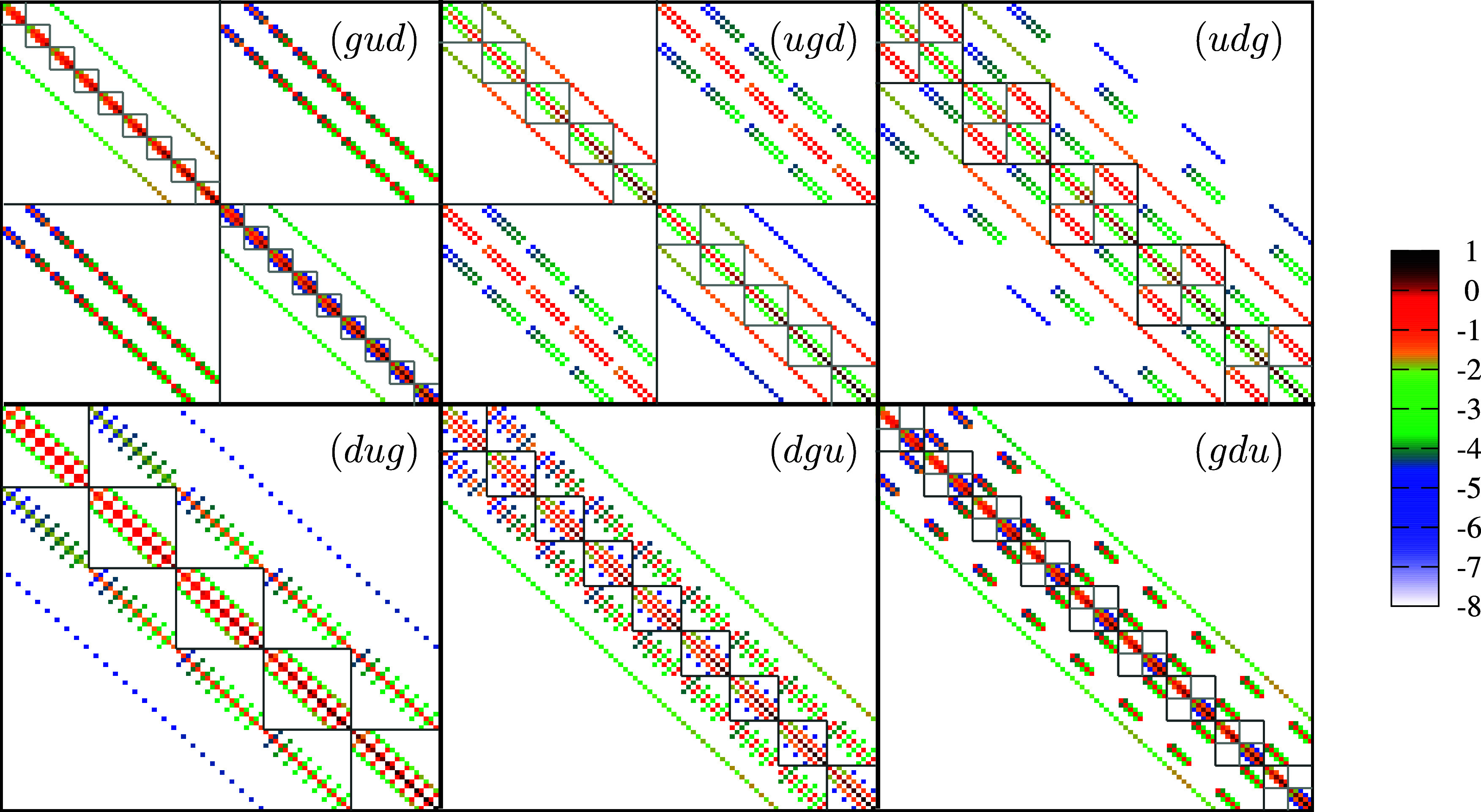
Structure of matrix *A* of the generalized model depending on the ordering of the basis, where the order is given by three letters *d*, *g*, and *u* representing the discrete states and vibrational modes *q*_*g*_ and *q*_*u*_. The first index changes the fastest. The size of the matrix elements is represented by the color in a logarithmic scale. The block-diagonal parts used for preconditioning are shown by the squares. The size of the basis for this visualization is *N*_*u*_ = 9, *N*_*g*_ = 5, for clarity, i.e., much smaller than in the actual calculations

For the solution of eq 30 in the original model, Estrada et al.^33^ devised a specially tailored method based on the block-tridiagonal structure of matrix *A*. Our aim in this paper is to develop a more general method capable of solving a larger class of models and test it both on the original model and on our generalization. Matrix *A* is large but sparse. From the character of the problem, it is also complex symmetric but not Hermitian. The structure of matrix *A* depends on the order of the basis vectors as illustrated in Figure A1 of the appendix for the generalized model.

## Krylov-Subspace Iteration Methods

The Krylov-subspace iteration methods (see Appendix for details) are ideally suited for the solution of the system of equations with sparse matrices, since they are based on expansion of the solution in the basis produced by repeated multiplication of the right-hand side *b* with matrix *A*. This matrix multiplication can be coded very efficiently (see Appendix). In this section, we test two methods: COCG (Conjugate orthogonal conjugate gradient) method and GMRES (generalized minimal residual) method well-known in numerical linear algebra. The following convergence test of the two iteration methods shows the convergence of the residuum *r*_*n*_ = *b* – *Ax*_*n*_ → 0 for the approximation *x*_*n*_ of the solution Ψ_α_ in the *n*th iteration. We discuss the convergence for different methods in terms of the number of iterations needed to obtain the residuum on the order of 10^–5^. The computational time is not given since it depends on details of both hardware and software tools used in the calculation, but as a rule of thumb, we can say that we need tens of minutes on desktop PC to get one 2D spectrum consisting of hundreds of energies in the case of the ECD86 model and its generalization but a computational cluster was needed for the calculation of the spectrum for CO_2_. In that case, solving the linear system took up to 10 h for one initial electron energy on a single CPU core and, in addition, a large amount of memory (∼20 GB) was necessary to store the matrix of the preconditioner. Note that the calculation can be easily parallelized over the initial energies since the claculations for different initial energies are independent of each other.

Before discussing the individual tests, we would like to point out that the essential ingredient of the iteration methods is the preconditioning. It is based on modification of matrix *A* → *M*^–1^*A*(*M*^*T*^)^–1^ (see Appendix), where *M* is easily invertible approximation to *A*. In the following, we discuss the preconditioning by matrix *M* consisting of the block-diagonal part of *A* for different ordering of basis vectors. We show small blocks *M*^*dg*^, *M*^*du*^, and *M*^*gu*^ of the sizes *N*_*u*_, *N*_*g*_, and 2, respectively, and the larger blocks *M*^*d*^, *M*^*g*^, and *M*^*u*^ of sizes *N*_*g*_*N*_*u*_, 2*N*_*u*_, and 2*N*_*g*_. For technical details, we refer again to the Appendix.

### Numerical Testing

We applied the methods GMRES and COCG described above to solve system (30) with matrix (32) for the original ECD86 model and for our generalization of the model. The performance of each method for different preconditioning is discussed separately for the two models in the next two paragraphs. The last paragraph also discusses the performance of the COCG method for a realistic model that describes inelastic electron scattering from the CO_2_ molecule. Note that the convergence properties (number of iterations) are independent of the size of the basis (*N*_*u*_, *N*_*g*_) used once the size is large enough to achieve the convergence, although the computational costs of the single iteration depend on these parameters.

#### Performance of the Methods for the ECD86 Model

**Figure 5:**
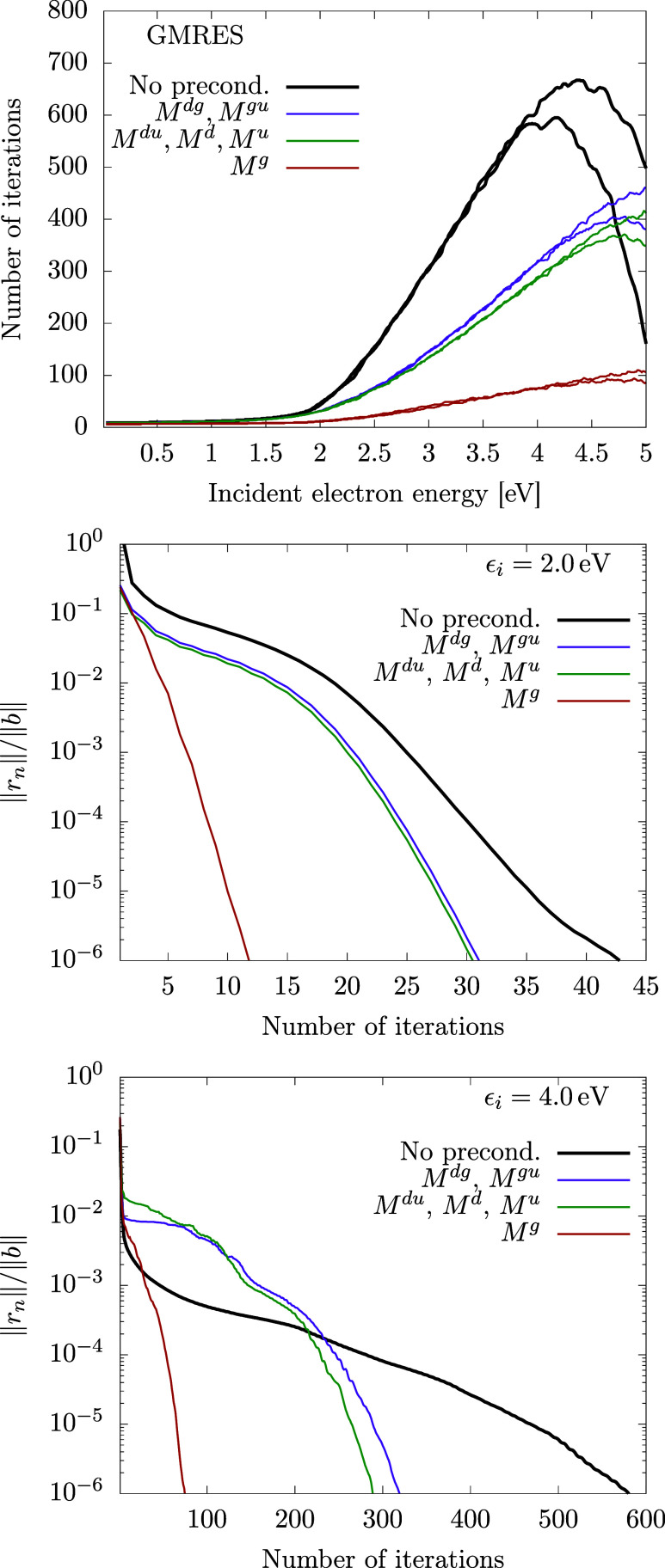
Convergence of the GMRES method for the model of Estrada et al.^33^ for various preconditioners. Number of iterations needed for each electron energy (top) and convergence of residuum for ϵ_*i*_ = 2 and 4 eV (bottom two panels). The top part shows results for both right-hand sides μ = *e*, *o* of the linear system; the bottom two parts display just μ = *e*.

**Figure 6:**
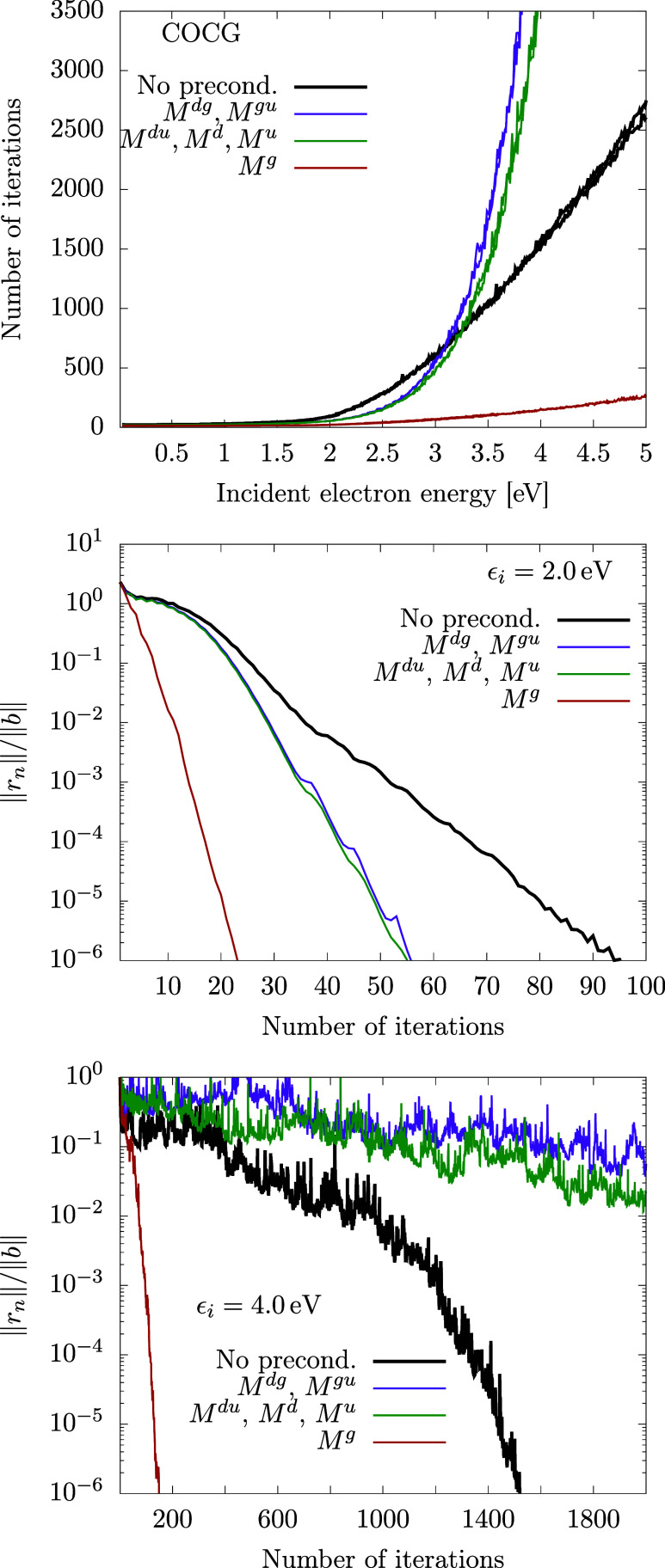
Convergence of the COCG method for the model of Estrada et al.;^33^ see also the caption of Figure 5 for details.

The performance is shown in Figures 5 and 6. Each of the figures is devoted to one of the methods comparing different preconditioning schemes. The top graph summarizes the number of iterations needed for convergence for all energies, and bottom two graphs demonstrate the decrease of the residuum norm for two selected energies ϵ_*i*_ = 2 and 4 eV. The different preconditioning methods are shown with different colors. The curves of the same color correspond to two different right-hand sides, μ_*i*_ = *o*, *e* in eq 30.

Let us first focus on the graphs at the top of Figure 5 showing the performance of the GMRES method. The method converges rather well (less than 700 iterations) even without any preconditioning. The convergence is extremely fast below 2 eV (several dozens of iterations) but gets slower above this energy with maximum around 4 eV. This is related to the spectrum of the anion. The electron with energy below 2 eV does not have enough energy to populate vibrational states of the anionic potential. The process of the electron scattering is therefore almost elastic, which means that the wave function is not much perturbed with respect to the initial state used for starting the iterations. Above this energy, the dynamics is much richer, which is reflected in the increased number of iterations needed to reach the converged wave function. For the most of the energies in the range of interest, the preconditioning reduces the number of iterations considerably. The least efficient preconditioning matrices *M*^*dg*^ and *M*^*gu*^ (overlapping curves in Figure 5) include only a diagonal portion of matrix *A* and are therefore numerically very cheap to implement. The preconditioner *M*^*du*^ includes also terms proportional to coupling constants κ_1_ and κ_2_. For the ECD86 model, there are no terms in matrix *A* added by increasing the size of the preconditioner to *M*^*d*^ and *M*^*u*^, and the convergence curves thus overlap for these three preconditioners. The best results are obtained with the preconditioning matrix *M*^*g*^, which has blocks of size 2*N*_*u*_ × 2*N*_*u*_ and includes terms proportional to λ in eq 18. The convergence of the residuum norm in the lower part of Figure 5 shows a difference in the behavior of different preconditioned methods. While the best method with the *M*^*g*^ preconditioner converges exponentionally for all energies, there is a kind of plateau in the other methods, and the iterations without preconditioning can even surpass some preconditioned methods.

The behavior of the COCG method (Figure 6) is different in several aspects. The overall number of iterations is approximately three times larger (for unpreconditioned iterations), but we have to keep in mind that the COCG method is much simpler with computational demands constant over the course of the iterations. For GMRES, the computational demands for one iteration grow quadratically with the number of iterations. The COCG method does not have the minimization property (40). This is reflected in the shape of the convergence curves (two bottom graphs in Figure 6). Unlike in similar curves for GMRES, here the residuum can locally grow, although in general it finally converges to zero. The efficiency of the different preconditioning schemes is similar like in GMRES, although for higher energies only the *M*^*g*^ preconditioner is useful.

#### Performance of the Methods for the Generalized Model

**Figure 7:**
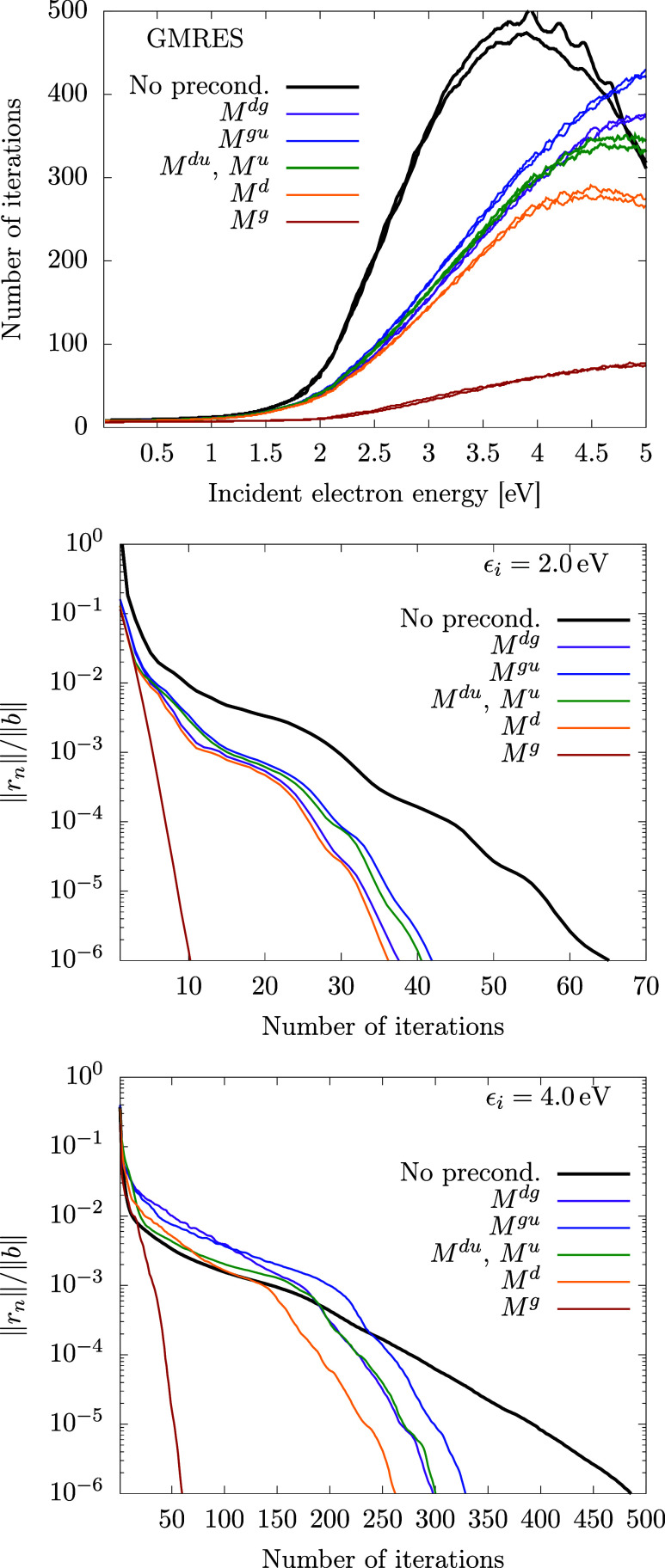
Convergence of the GMRES method for the generalized model; see also the caption of Figure 5 for details.

The generalized model has a more complicated structure (27) of the level-shift operator *F*(*E*), which is reflected in a more complicated structure of matrix *A*; see Figure A1. Surprisingly, the iteration methods converge more quickly with this matrix. There are no clear criteria rigorously relating the structure of the matrix to the speed of convergence. We believe that the faster convergence here may be related to the fact that operator *F* in the generalized model increases diagonal elements of matrix *A*. Apart from a little bit faster convergence, the graphs in Figure 7 for the GMRES method in the new model look qualitatively similar to those for the ECD86 model. The norm of the residuum is monotonously decreasing for all methods, and the preconditioner *M*^*g*^ is again the most efficient. The individual preconditioners now lead to different convergence rates because all choices of the diagonal blocks are distinct for the richer structure of *A*. The exception is the equivalence of *M*^*du*^ and *M*^*u*^ preconditioning. This can be nicely understood from the structure of matrix *A* depicted in Figure A1. We see that the large and small black diagonal boxes in the bottom right matrix differ by a blank area of zero matrix elements.

**Figure 8:**
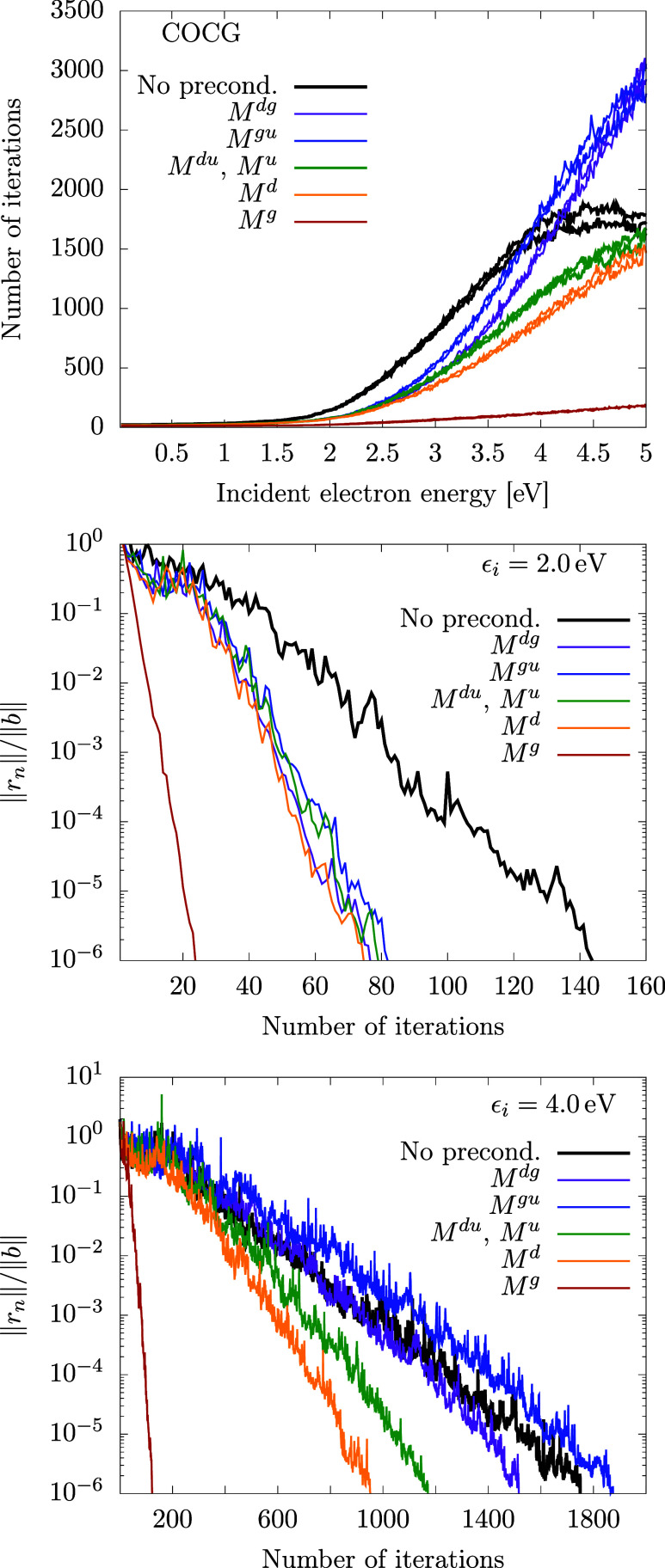
Convergence of the COCG method for the generalized model; see also the caption of Figure 5 for details.

The faster convergence for the new model is even more apparent for the COCG method in Figure 8. Now, all preconditioning schemes except for *M*^*dg*^ and *M*^*gu*^ are faster than direct iterations.

To conclude the numerical experiment section, we add a few notes on the implementation. Even without utilizing the structure of matrix *A*, we have got by 1 order of magnitude faster calculation of the spectra utilizing the Krylov-subspace iteration methods as compared to a direct solver. Optimizing the matrix-vector multiplication using the structure of matrix *A* explained at the beginning of the Appendix leads to another order of magnitude speed up. From the previous examples, we see that the proper choice of preconditioning leads to the decrease of the number of iterations needed for convergence by another 1 order of magnitude for both models and both methods.

#### Performance for the Model of *e*^–^ + CO_2_

In the final part of this section, we discuss our earlier work^34−36^ on the electron collisions with the carbon dioxide (CO_2_) molecule in the context of the present paper. The vibronic coupling model for the *e* + CO_2_ system^35^ follows the general approach presented here in Section “Generalized Model with Vibronic Coupling with Continuum States”; however, the model is more complex. We considered the nuclear motion within the full four-dimensional vibrational space in combination with three electronic states (^2^Σ_*g*_^+^ virtual state and two components of the ^2^Π_*u*_ shape resonance), which are coupled upon bending of the molecule. The Hamiltonian is thus a 3 × 3 matrix in the electronic space and we did not restrict its elements only to the first order in the normal coordinates (some of the elements were expanded up to the fourth order). Additionally, the three discrete states were coupled to four electron partial waves. The vibrational dynamics is described analogously to the scheme given in Section “Numerical Representation of the Dynamics” but there are four vibrational indices instead of two. The vibrational basis was constructed from products of eigenfunctions of 1D harmonic oscillators for symmetric and stretching modes and eigenfunctions of the 2D harmonic oscillator expressed in polar coordinates for the two-dimensional bending mode.

**Figure 9:**
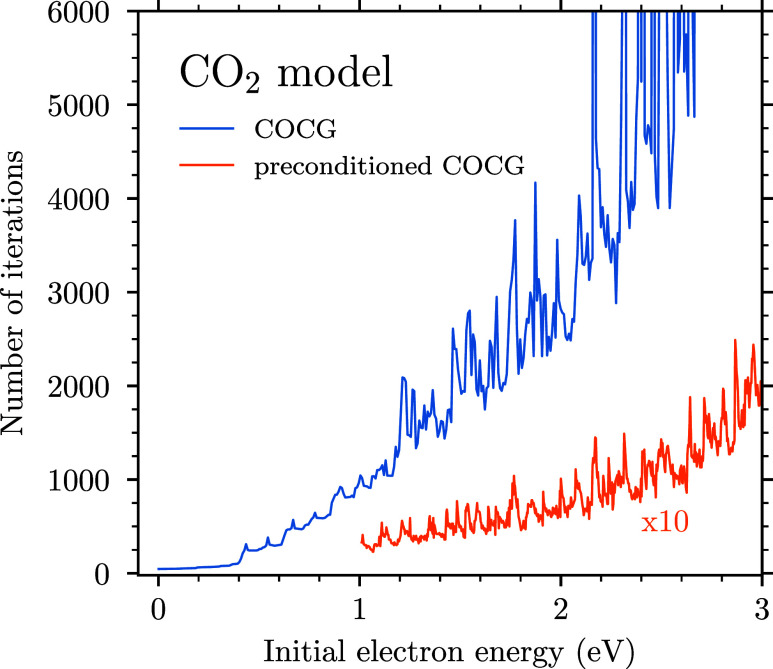
Number of iterations needed to solve the Schrödinger equation for the *e* + CO_2_ system using the COCG method without and with preconditioning. In the latter case, the curve is multiplied by a factor of 10.

Using the COCG method without any preconditioning, the number of iterations needed to reach the convergence with the stopping criterion of 10^–3^ (sufficient to obtain converged cross sections) rapidly grows with the electron energy; see Figure 9. For energies above 3 eV, even 2 × 10^5^ iterations were insufficient to reach the convergence; therefore, a suitable preconditioning is essential.

The slow rate of convergence or no convergence at all is caused by the coupling of the discrete states through the bending mode. The stretching modes do not affect the convergence much since we found that the COCG method converges badly even for the case where we did not consider the stretching modes.^2^ Thus, taking a block-diagonal preconditioner where blocks contain discrete states and two-dimensional bending was a natural choice. Such a preconditioner is analogous to the preconditioner *M*^*g*^ that performs the best for the ECD86 model and its generalization. In the case of CO_2_, around 200 iterations were sufficient to reach the convergence for an initial electron energy of 3 eV; see Figure 9.

## Discussion of Resulting Spectra for Test Models

It is not the purpose of this paper to study in detail the calculated spectra and their interpretation. This will require a detailed analysis of the final-state distribution and shape of the individual components of the wave function in the coordinate representation and its relation to the shape of potentials and also study of the dependence of the results on the model parameters. It is a quite voluminous work that deserves a separate paper. We also identify specific molecules that can be treated with the model of the current setup or a proper generalization. We already published the generalization of the model^35^ needed to describe the resulting spectra for the CO_2_ molecule^34^ and performed the detailed analysis^36^ including the final-state distribution, the wave functions, and decomposition of spectra due to the contribution of components of different symmetry.

In the following, we show and briefly describe the 2D spectra for the ECD86 model (which were not the subject of the original paper) and for our new generalization of the model. We also separate the contribution of the two right-hand sides in eq 30 corresponding to the gerade and ungerade symmetry, and finally we study the energy dependence of the cross section for excitation of the fundamental modes.

### 2D Spectrum for the ECD86 Model

**Figure 10:**
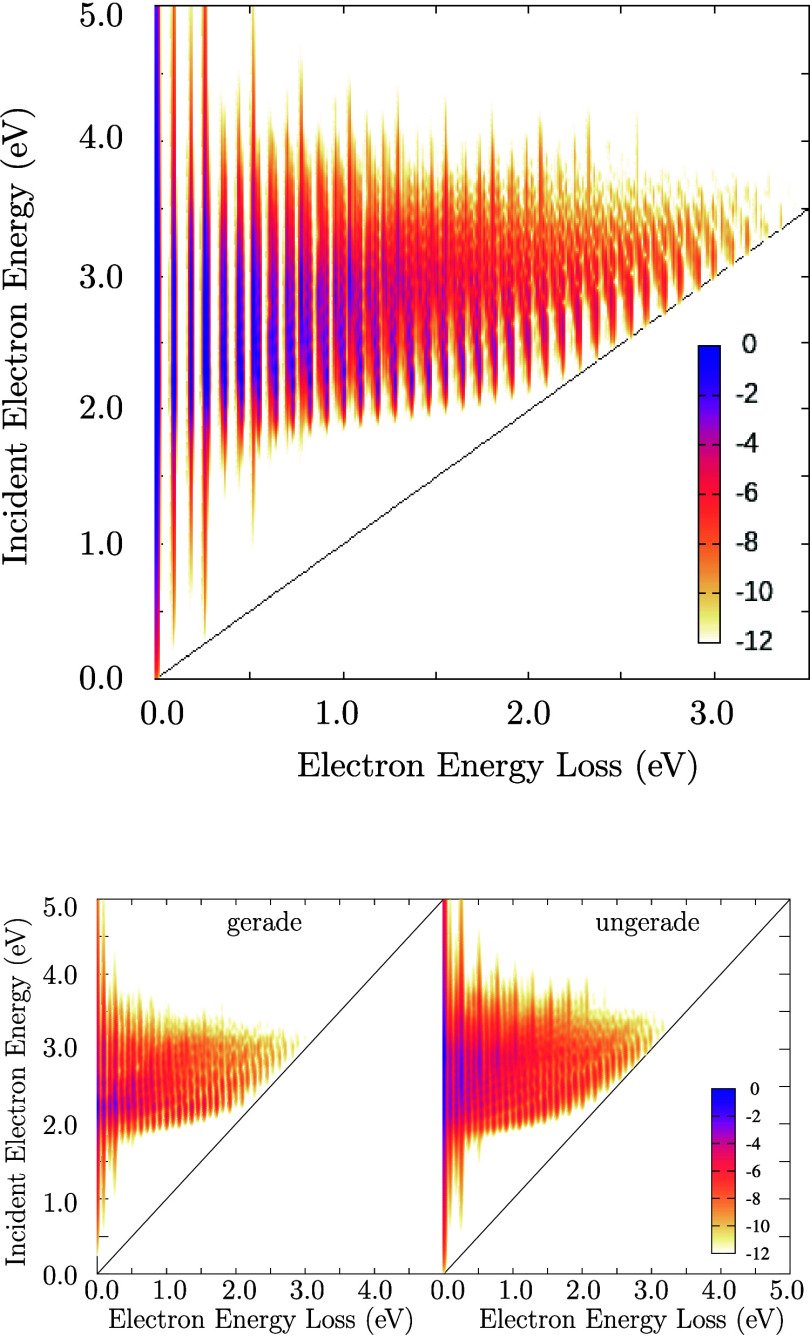
2D electron energy-loss spectrum for the ECD86 model (top) and its decomposition to the gerade and ungerade symmetries (bottom). The intensity of spectrum is shown in a logarithmic scale.

The calculated 2D spectrum for the ECD86 model is shown in Figure 10. The intensity given by eq 17 is plotted as a function of both energy loss Δϵ and initial electron energy ϵ_*i*_ in a color logarithmic scale. It is fully converged result, i.e. it is independent of the method used to calculate it. Interestingly enough, the spectrum is qualitatively quite similar to the 2D spectrum for the CO_2_ molecule^24,34^ in the region of the Π_*u*_ resonance. The bulk of the spectrum is located at energies of the incident electron between 2 and 4 eV. This is a consequence of the shape of the anion potential manifold (Figures 1 and 2) and its location relative to the potential of the neutral molecule. The understanding of the detailed shape is not trivial. For small electron energy losses, the spectrum is discretized by vibrational frequencies whose ratio is approximately 3:1. But since this ratio is not exact, the spectrum becomes quasi-continuous for energies above 1 eV. At the same time, we see that there is some selection mechanism that singles out narrower structures close to the diagonal threshold line. There are also diagonal rays appearing in the structure of the spectrum (better apparent in the decomposition of the spectrum according to symmetries). Both of these features were present in the case of CO_2_, where we performed the detailed analysis.^36^

### 2D Spectrum for a New Model

**Figure 11:**
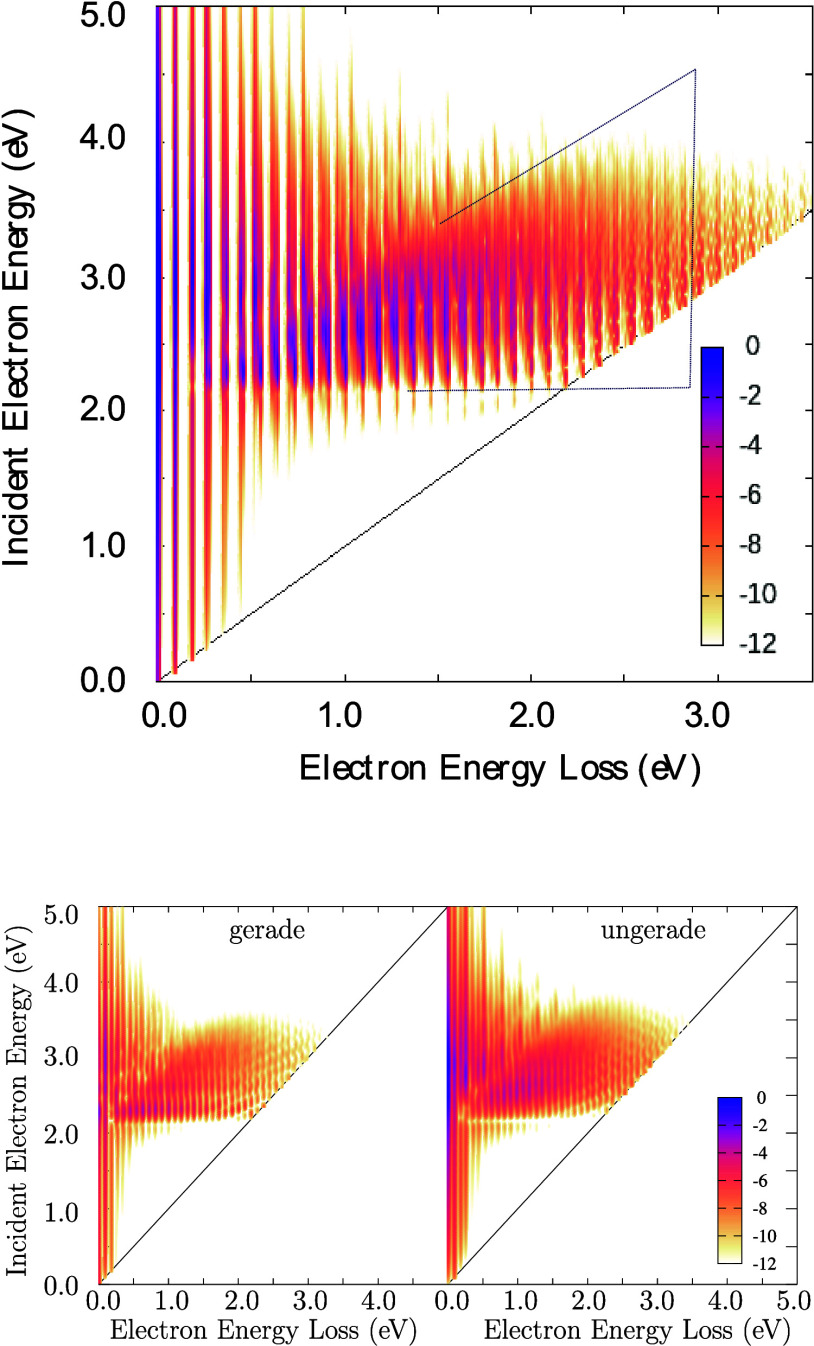
Same data as in Figure 10 but for the new model. The black dotted lines in the top figure indicate a triangle containing the feature discussed in the text. This feature extends between the horizontal and diagonal lines almost all the way toward zero energy loss.

We proposed the new model above to consistently introduce the vibronic coupling in the level-shift operator in the ECD86 model and to test the iteration schemes to solve the dynamics in this model. The choice of the model parameters was guided by our experience with the diatomic molecules but apart from that, the choice is completely random. To our surprise, the resulting spectrum (see Figure 11) has a quite interesting intricate structure, which is furthermore similar to experimental data for some molecules, like benzene and its derivatives.^52^ Particularly, we are talking about the wedge-shaped structure marked in Figure 11. The origin of this structure is not clear and since it is quite common in experimental data, we will dedicate the future study to this phenomenon. It indicates some selection mechanism in the dynamics that forces the system to skip through a region with small energy losses to large losses.^52^

### Vibrational Excitation Integral Cross Sections

**Figure 12:**
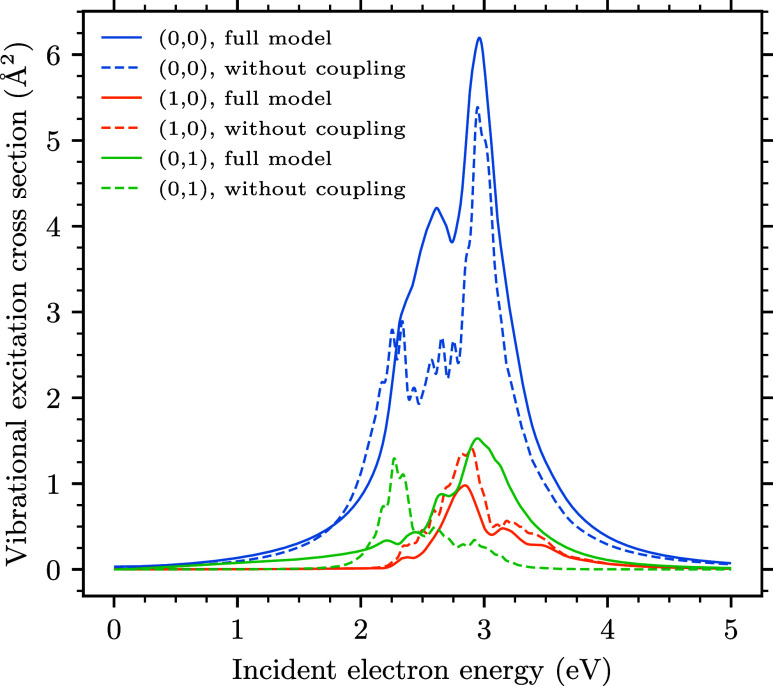
Integral cross sections for the elastic channel and the excitation of the two vibrational modes. The dashed curves are the data for a model with the off-diagonal coupling though the continuum switched off.

To better understand the role of the conical intersection in the vibrational excitation process, we show the individual cross sections σ_*v*_*f*_ ← *v*_*i*__ for the elastic channel *v*_*f*_ = (0, 0) and for the excitation of one quantum of the two modes, *v*_*f*_ = (1, 0) and (0, 1) as the function of the initial electron energy ϵ_*i*_ in Figure 12. Further, we tested the effect of the off-diagonal couplings *F*_12_ and *F*_21_ of the discrete states through the continuum on the dynamics. The dashed curves in Figure 12 are the results of the calculation where these two terms were switched off by setting the parameters *a*_1_^*o*^ and *a*_2_^*e*^ (see Table 2) to zero. First of all, we observe that the nonlocal coupling has a little effect on the elastic cross section except for the energies in the vicinity of the position of the conical intersection (around 2.5 eV), where the presence of this additional coupling terms increases the cross section by ∼30%.

The data nicely reflect the fact that the discussed coupling terms are proportional to the coordinate *q*_*u*_ of the nontotally symmetric mode and thus have a larger influence on the excitation of the ungerade mode *v*_*f*_ = (0,1). The cross section from the fully coupled model for this state peaks around ϵ_*i*_ = 3.0 eV, while the dashed curve exhibits a triple maximum close to ϵ_*i*_ = 2.3 eV, which can be understood by examining the width of the adiabatic states. The width of the upper branch of the conical intersection gets wider as the coordinate *q*_*u*_ increases (see the left panel of Figure 3), while the width nearly vanishes in the lower branch close to the intersection. This enhances the cross section of the (0,1) excitation above the intersection energy of 2.5 eV and suppresses it below this point. The width on the lower branch gets broader again close to the crossing with the potential energy surface of the neutral state, which is reflected by the fact that the cross section for the full calculation again exceeds the dashed curve at small energies. Also note that the potential energy curves of the limited model (not shown) are very similar to those of the original ECD86 model (see Figures 1 and 2). The peaks close to ϵ_*i*_ = 2.3 eV in the dashed blue curve can thus be traced to the minima in the potential energy surface of the anion similar to those in the left panel of Figure 1.

In contrast to this behavior, the cross section for the excitation of the symmetric mode (1,0) is not very sensitive to the presence of the additional terms, and both the solid and dashed curves follow the same behavior with similar magnitudes.

We expect that the off-diagonal coupling will be even more important for the behavior of the differential cross sections because the involvement of different partial-wave components of the electron continuum in the matrix of the coupling amplitudes *V*_ϵ_ is subjected to selection rules, as discussed above. Each partial wave contributes to the angular dependence of the differential cross section in a different way as we discussed in detail for the CO_2_ molecule.^34−36^ In this series of papers, we found that the detailed analysis of the spectra and differential cross sections requires also the investigation of the wave functions and the variation of various parameters of the model. This goes beyond the scope of this paper, which focuses on the derivation and numerical solution of the model. However, it will be the subject of a future study that will focus on the interpretation of 2D EELS spectroscopy in terms of the potential energy curves of the discrete states and their widths and couplings to different continuum channels.

## Conclusions

We derived a generalization of the model of conical intersection in electronic continuum proposed originally by Estrada et al.^33^ by including terms linear in the vibrational coordinates also in the term that couples the two discrete states of the original model to two partial waves of the electronic continuum. The generalization thus produces linear and quadratic terms in the nonlocal level-shift operator *F*(*E*) that describes the dynamics of the vibrational excitation of the molecule by collision with an electron. The dependence on the nontotally symmetric vibrational coordinate also allows indirect coupling of the two discrete states of different symmetries through the electron continuum.

To solve the vibronic dynamics, we implemented two Krylov-subspace iteration methods, GMRES and COCG, which are ideally suited for this kind of model that produces a sparse-matrix representation of the Schrödinger equation. Essential for the efficiency of these methods is the choice of a suitable preconditioner. We tested both methods in detail, and we provided guidelines for the choice of the preconditioner.

We also briefly analyzed the resulting two-dimensional electron energy-loss spectra and the energy dependence of the integral cross sections. These spectra exhibit a surprisingly complex structure, and their full understanding will require a more detailed study with the inspection of the wave functions and perhaps also time-dependent calculations. Here, we show that the separation of the contribution of different irreducible representations to the dynamics disentangles the complex pattern to some degree. Another insight was gained by inspection of the energy dependence of cross sections for some final states. The elastic cross section and the excitation of the symmetric mode are not very sensitive to the presence of the indirect coupling through the continuum except in the vicinity of the energy of the conical intersection. On the other hand, the excitation of the nontotally symmetric vibrational mode is strongly influenced by this coupling. This aspect may be important for many molecules with a nontrivial symmetry group.

We believe that the methods tested here can be used for more complicated molecules to get a better understanding of the 2D electron energy-loss spectroscopy. We plan a more extensive parameter study to obtain a deeper understanding of the results. The proposed method is conceptually simple and can be further generalized in a straightforward way to include more anion states, more vibrational degrees of freedom, and higher-order polynomial functions. A more challenging generalization will be needed to also include dissociative channels and anharmonicity of the neutral molecule.

## Appendix

### Details of the Computational Methods

The Krylov-subspace iteration methods (see for example monographs^53,54^) are well-suited for solving eq 30. The main idea of all of the Krylov-subspace methods is that they solve a linear system

34

iteratively producing a sequence *x*_0_, *x*_1_, *x*_2_, ···, *x*_*n*_ of approximations of the solution vector *x* in the Krylov-subspace, i.e., in the space

35

where *r*_0_ = *b* – *Ax*_0_ denotes the initial residual vector. The methods differ by the definition of the ”ideal” approximation of the solution *x*_*n*_ within the Krylov subspace and usually proceed by application of simple recursive formulas. To produce the Krylov subspace, we only need to implement the matrix multiplication *Aw* for an arbitrary vector *w*. The choice of the harmonic basis is very convenient for the implementation of this matrix multiplication. From eqs 13, 18, and 27, we can see that the multiplication by matrix *A* can be decomposed to successive multiplications by energy-dependent diagonal factors, for example

36

37

and by operators of the coordinates *q*_*g*_ and *q*_*u*_

38

These operations can be implemented very efficiently. Note that all energy factors and square roots can be precalculated and stored before starting the iteration process. Compared to a matrix-vector multiplication, which requires *O*(*N*^2^) operations for full matrices, the above procedure requires only *O*(*N*) operations (with the operation count being approximately three times larger for the generalized model due to a more complicated structure of the operator *F*). The efficiency of the method of solution of eq 30 is then given by the rate of convergence of the sequence *x*_*n*_ to the solution, which is judged by monitoring the size of the norm of the residuum ∥*r*_*n*_∥ = ∥*b* – *Ax*_*n*_∥. In the following tests, we stop the iterations when the value ∥*r*_*n*_∥ < 10^–5^ ∥*b*∥ is reached.

### Methods of Interest

Saad and Schultz^55^ developed the generalized minimal residual (GMRES) method, one of the most widely used Krylov-subspace methods. This method constructs an orthonormal basis *v*_1_, *v*_2_, ···, *v*_*n*_ of the *n*th Krylov subspace  using the Arnoldi algorithm that can be written in the matrix form as

39

where  is an upper Hessenberg matrix. The approximation *x*_*n*_ of the solution in each step is given by the condition that the residual vector *r*_*n*_ = *b* – *Ax*_*n*_ satisfies the optimality property

40

If we write *x*_*n*_ = *x*_0_ + *V*_*n*_*y*_*n*_, this condition leads to the (*n* + 1) × *n* least-square problem for *y*_*n*_

41

which has to be solved in every iteration.

An advantage of the GMRES method is that it can be used for any matrix *A* with no other special properties required than the regularity. On the other hand, each new basis vector at every iteration step has to be orthogonalized to all previous vectors. Thus, the size of the matrix *V*_*n*_ grows during the iteration process and if the method does not converge quickly, the storage space and the time needed for each step grow significantly. Note that matrix A of our system of equations is complex-symmetric (i.e. not Hermitian). For this reason, the conjugate gradient method (which, unlike the GMRES method, uses short-term recurrences to construct the basis of the Krylov subspace, making it much less computationally demanding) cannot be used to solve it. It is known^56^ that for nonnormal matrices, it is not possible to define an “optimal” iterative process (i.e. a process that minimizes the residual or certain norm of the error over the Krylov subspace) that constructs the basis of Krylov subspace using short-term recurrences.^54^ For complex symmetric matrices, van der Vorst and Mellisen^57^ presented an alternative way to define an iterative process based on three-term recurrences and derived the conjugate orthogonal conjugate gradient (COCG) method. Setting *v*_0_ = *r*_0_ = *b*–*Ax*_0_, the basis of the Krylov subspace is constructed using the recursive formula

42

where α_*n*_ and β_*n*_ follow from the conditions ⟨*v*_*n*+1_|*v*_*n*_⟩_*S*_ = 0 and ⟨*v*_*n*+1_|*v*_*n*–1_⟩_*S*_ = 0. These conditions are analogous to those that define the conjugate gradient method, in which, however, we have replaced the standard scalar product with the symmetrized bilinear form

43

Note that the complex conjugation in the left argument cancels the complex conjugation in the standard definition of the scalar product. The bilinear form ⟨·|·⟩_*S*_ is not therefore positive-definite but it preserves the symmetry of matrix *A*. This process ensures that vectors *v*_0_, *v*_1_, ···, *v*_*n*_ satisfy the conjugate orthogonality property (vectors  are conjugate-orthogonal if ⟨*a*|*b*⟩_*S*_ = 0). The whole iterative process is thus analogous to the conjugate gradient method but the convergence after *N* steps (*N* being the dimension of matrix *A*) is not guaranteed in the exact arithmetic. In addition, the iterations do not have to converge at all since the symmetrized product can be zero (or very small) even for nonzero vectors. In practice, however, the convergence is usually achieved. Moreover (especially with proper preconditioning), it is often rather fast.

### Preconditioning

In this paper, by preconditioning we understand the transformation of the original linear system *Ax* = *b* into an equivalent problem (see for example the paper by Saad^53^)

44

with a regular matrix *M*. This way, the preconditioning preserves the symmetry of matrix *A*. As a rule of thumb, a good preconditioner *M* is represented by some fast invertible approximation of the original matrix *A*, but there are no exact guidelines for choosing the ideal matrix *M* ensuring a fast convergence for the transformed problem. There is a large variety of preconditioners known in the literature but usually they are proposed for specific problems with matrices *A* having some special properties. We tested some of the preconditioning options but we found that the most simple methods work best for the present problem;^58,59^ see footnote^3^. In the following, we discuss different possibilities of block-diagonal preconditioning. For this choice, we define the matrix *M* as a block-diagonal section of the original matrix *A*. Depending on the structure of the blocks, the individual blocks can either be inverted directly to construct *M*^–1^ or a banded structure of the blocks can be used. The exact structure of the blocks depends on the ordering of the basis (see Figure A1). We thus define three different preconditioners *M*^*dg*^, *M*^*gu*^, and *M*^*du*^ with small diagonal blocks of sizes *N*_*u*_ × *N*_*u*_, 2 × 2, and *N*_*g*_ × *N*_*g*_, respectively, and three preconditioners *M*^*d*^, *M*^*g*^, and *M*^*u*^ with large blocks of sizes *N*_*g*_*N*_*u*_ × *N*_*g*_*N*_*u*_, 2*N*_*u*_ × 2*N*_*u*_, and 2*N*_*g*_ × 2*N*_*g*_. To be more specific, in the (*dug*) ordering of the basis functions, the small-block preconditioning matrix *M*^*ug*^ has *N*_*u*_*N*_*g*_ blocks *M*^(*n*_*u*_,*n*_*g*_)^ of the size 2 × 2 with the matrix elements

45

and the large-block preconditioning matrix *M*^*g*^ has *N*_*g*_ blocks *M*^(*n*_*g*_)^ of the size 2*N*_*u*_ × 2*N*_*u*_ with the matrix elements

46

To apply the preconditioning, we need to act with *M*^–1^ on a vector *w* with components *w*_*d*,*n*_*g*_,*n*_*u*__. This can be done block by block; for example, to apply the preconditioner *M*^*u*^, we invert each block *M*^(*n*_*u*_)^ using the *LL*^*T*^ (or *LDL*^*T*^) decomposition and act on the section of *w*_*d*,*n*_*g*_,*n*_*u*__ vector for this fixed *n*_*u*_

47

This is repeated for each *n*_*u*_. Note that the inverting of the preconditioning matrices *M*^(*n*_*u*_)^ should be done just once and stored before starting iterations. Moreover, in practice, the *L* (and *D*) matrix is stored in memory instead of *M*^(*n*_*u*_)–1^.

## Footnotes

1 Note that we consider *V*_*d*ϵ_^μ^ to be real quantities. This is a reasonable assumption since the coupling can be made real for a single *q⃗* by phase conventions and the dependence on *q⃗* is supposed to be weak (diabaticity of the basis).

2 We can easily freeze a vibrational mode by considering only the ground state as the basis within this mode and setting all relevant model parameters to zero.
